# The patterns of antisense long non-coding RNAs regulating corresponding sense genes in human cancers

**DOI:** 10.7150/jca.49067

**Published:** 2021-01-01

**Authors:** Min Zhou, Xingjun Guo, Min Wang, Renyi Qin

**Affiliations:** Department of Biliary-Pancreatic Surgery, Affiliated Tongji Hospital, Tongji Medical College, Huazhong University of Science and Technology, Wuhan, China.

**Keywords:** antisense long non-coding RNAs, sense genes, human cancers, regulation patterns

## Abstract

For decades researches of genomic transcription of all kinds of species have demonstrated that the important role of Long non-coding RNAs (LncRNAs) in whole process of life entity has been more and more attached. Owing to constant developing of advanced technology, especially the emerge of next generation sequencing, researchers could explore further in the depth and breadth of LncRNAs. Given that the unique RNA loci location with its corresponding sense gene, antisense long noncoding RNAs (AS-lncRNAs), which are one of the main categories of LncRNAs classification, would have existed an identified close connection between them in a natural physiological state. This review characterizes the patterns of regulation between AS-lncRNAs and corresponding sense genes during the process of cancer progression in human, with emphases on the regular modulation ways of the potential molecular mechanism of AS-lncRNAs and the summary of underlying treatment targets in human cancers.

## Introduction

Long non-coding RNAs (LncRNAs) are one category of uncapable encoding RNAs, with length more than 200 nucleotides. LncRNAs have some common specificities, for example a methyl guanosine cap at their 5' end and a polyA tail at the 3' end (polyA), comparing with encoding mRNAs which also transcribed by RNA polymerase II (Pol II) 1, 2. Owing to the high-throughput sequencing technologies emerging, we could explore the non-coding genomes with unprecedented speed and massive scale 3. According to the location of LncRNAs' loci biotypes related with protein-coding genes, there is a widely accepted classification which contains : 1) antisense LncRNAs, which intersect with any exons of protein-coding locus on the other strand. 2) sense LncRNAs overlapping the coding transcripts are embedded in one of the introns on the same strand, or reside in introns without touching any exons. 3) LincRNAs belonging to the group of transcripts existing in intergenic sites. 4) Others which don't belong to any categories still without an open reading frame (ORF) 4.

Previous researches indicated that the number of disorders in protein-coding genes and LncRNAs are approximately equal in thousands of cancer tissues samples, while intriguingly the 60% of LncRNAs show the strict specificities to only one type of tumor sample 5. Though LncRNAs may be not as conserved as mRNAs, they indeed show strong cell line, tissue and organ specificity. Abundant evidences show that aberrantly expressed LncRNAs has participated in cancer progression as an indispensable role. PCA3, PRNCR1and PCGEM1 are lncRNAs exclusively involved in the progression of prostate cancer, what is more, PCA3 in urine could been a mature clinical tool for diagnosis of prostate malignant tumor 6. The overexpression of LncRNA MALAT1 has a very close relationship with early-stage lung adenocarcinoma and can be the biomarker for the poor prognosis 6. LncRNA NORAD (Noncoding RNA Activated by DNA damage) once was found to involve in the sequestration of the promoting factor PUMILIO proteins in DNA damage, and to participate in breast cancer as an oncogene by regulating TGF-β signaling pathway 7, 8. No matter in the field of early diagnosis or precise treatment, LncRNA has a tremendous value in the research of human cancers and is bound to play the vital roles.

Given that the unique RNA loci location with its corresponding sense gene, AS-lncRNAs, as one of the categories of classification of LncRNAs, would have an identified close connection with homologous sense genes in a natural physiological state. The model would have been ubiquitous in caner progression. For example, ZEB1-AS1 promoted proliferation and migration of prostate cancer cells by binding and recruiting histone methyltransferase MLL1 to the promoter region of ZEB1, inducing H3K4me3 modification therein, and activating ZEB1 transcription 9. This review aims to find the regular patterns of AS-lncRNAs regulating corresponding sense genes in human cancer, and to provide a study approach to exploit methods of early-diagnosis and new gene treatments.

## Post-transcription Regulation Patterns of Antisense LncRNAs in Cytoplasm

The anatomical properties of the gene loci may imply the regulating potential and mode 10; And it couldn't be more suitable way to describe the function of AS-lncRNAs which is utilizing the post-transcription ability to regulate the conjugate protein-coding genes in accordance with the location of cytoplasm. AS-lncRNA can directly and indirectly interact with the mRNA of complementary protein-coding gene and regulate its stability and transcription efficiency.

## AS-lncRNAs act as “stabilization factor” to form double strands to avoid degradation of mRNAs

**Figure 1:**
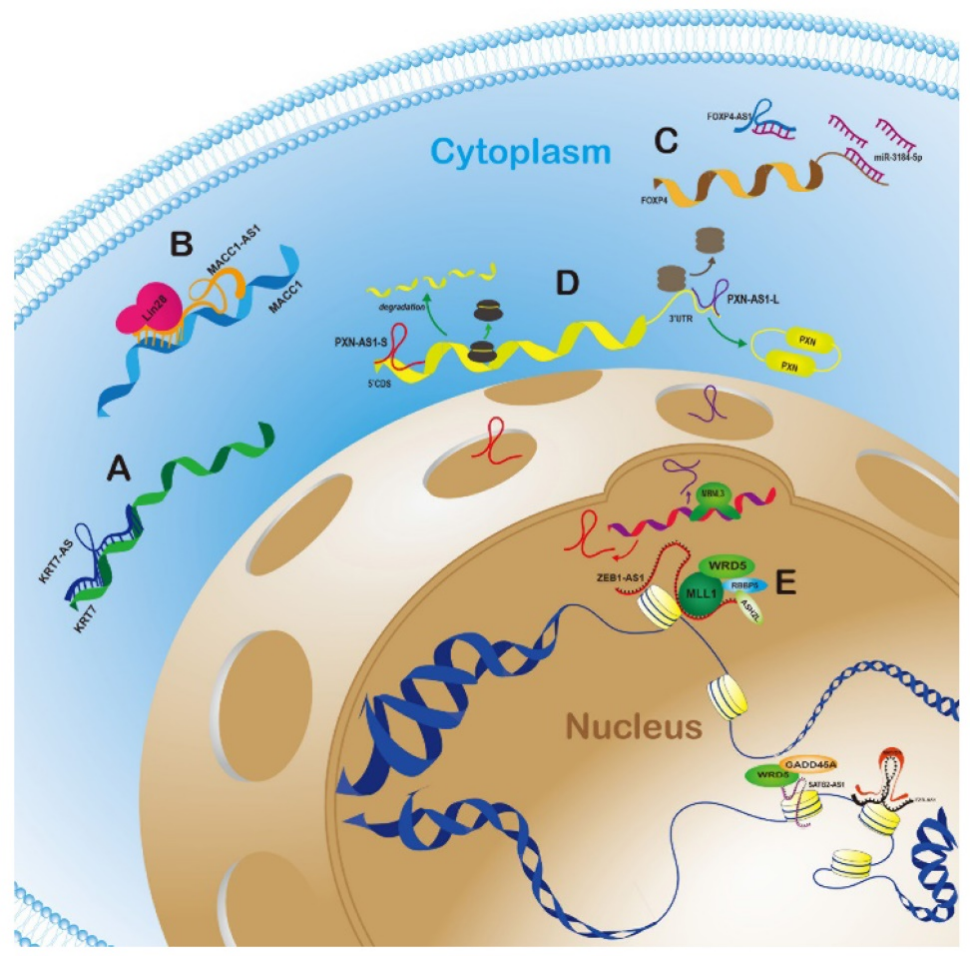
** Graphic mechanisms illustration of typical antisense lncRNA-sense gene regulatory modes in cytoplasm and nucleus.** A.AS-lncRNAs can directly bind to and stabilize sense genes mRNAs in cytoplasm; B. AS-lncRNAs can recruit other proteins to prolong the half-life of target mRNAs in cytoplasm; C. The indirect regulation way is that AS-lncRNAs counted as “ceRNA” sequesters sense mRNAs by competitively binding miRNA; D. After alternatively spliced, two variants of AS-lncRNAs can exert bidirectional function on opposite genes in cytoplasm; E. The mainstream mode in nucleus is that AS-lncRNAs regulate the methylation states of sense genes promoter region and activate or inactivate target genes.

Zhao et al. made use of next-generation sequencing analysis to explore the most prominent diverse expression AS-lncRNAs in GC (gastric cancer) tissues and cell lines, and focused on RP3-416H24.1. UCSC genome browser illustrated that RP3-416H24.1 was a single antisense RNA that is transcribed from the opposite strand of the Homo sapiens keratin 7, type II (KRT7) locus and was noted as KRT7-AS. KRT7-AS was principally recognized in nucleus and positively related with the expression of KRT7 in mRNA and protein level. KRT7-AS can affect the mRNA of KRT7 stability by RNA polymerase II inhibitor assay, and was speculated to form complementary complex with the mRNA of KRT7 to utilize the “GUARD” role (Fig. 1A). Furthermore, the increasing KRT7 protein expression in GC cells after transfection with overlapping-region (OL)-only vector and full-length (FL) vector implied that only including overlapping region, KRT7-AS can protect the KRT7 mRNA from degradation, which verified the double strand structure formation of AS-LncRNA and mRNA. Intriguingly, KRT7-AS was found in nucleus mainly, which may translocate from nucleus to cytoplasm and escort the mRNA of KRT7 until translation. KRT7-AS and KRT7 synergistically acted as oncogenes to promotes the progression of GC in the form of RNA-RNA duplex 11. Chen et al. further illustrated that Fusobacterium nucleatum(Fn) could upregulate the expression of KRT7-AS/KRT7 to facilitate the migration and metastasis of colorectal cancer in the level of the microorganism environment 12. The dysfunction of KRT7-AS along with KRT7 in gastrointestinal cancers indicated the tissue specificity of them, which would provide a new method in gene and microorganism level oncotherapy.

MUC5B is a member of the mucin (MUC) family, which are highly glycosylated macromolecular components of mucus secretions 13; And its overexpression has positive correlation with early post-operation metastasis and poor survival in the patients with lung adenocarcinoma 14, 15. MUC5B-AS1 was the cognate antisense transcript of MUC5B, which were synchronously influenced each other in the level of expression in lung adenocarcinoma. As a novel lncRNA, the aberrant expression of MUC5B-AS1 was firstly detected in lung cancer. Excluding the same transcription factor's effect on them both, Yuan et al. were interested in the complementary nucleotide base and figured out that the OL region was the necessary functional element to protect the mRNA from digested by RNase. Moreover, biotin-labeled MUC5B-AS1 pull-down assays verified the direct binding relation between MUC5B-AS1 and MUC5B mRNA. Breaking the mutual stabilizing function between MUC5B-AS1 and MUC5B may provide a novel chemotherapy strategy to treat the lung adenocarcinoma 16.

Under the metabolic stress condition, MACC1-AS1 and MACC1 was induced and promoted the GC progression by enhancing metabolic plasticity 17. Without the ability of regulating the promoter region and stabilizing the protein of MACC1, MACC1-AS1 could directly bind and protect the mRNA of MACC1 from degradation by mRNA degradation assays and RNA pull-down assays respectively (Fig. 1B). However, the bypass function of MACC1-AS1 in stabilization of MACC1 was that AMPK activated by overexpression of MACC1-AS1 translocated the Lin28 from nucleus to the cytoplasm to directly bind the mRNA of MACC1 and prevent it from degradation. In general, MACC1-AS1 can regulate the stability of the mRNA of MACC1 in direct and indirect ways to promote GC tumorigenesis under metabolic stress 18. Even though MACC1-AS1 was involve in the progression of gastric cancer, pancreatic cancer, hepatocellular carcinoma, lung cancer, cervical squamous cell carcinoma, nasopharyngeal carcinoma and glioma, the dysfunction pattern of MACC1-AS1/ MACC1 only existed in some certain cancers, which provided more possibility of chasing the precise target of oncotherapy.

## AS-lncRNAs act as “scaffolds” to change the half-life of mRNAs

Cancer-testis (CT) genes are a cluster of genes that finitely expresses in germ cells and under some certain circumstance aberrantly expresses in tumors 19. LIN28B 20, a novel CT gene, promotes the proliferation and metastasis of lung adenocarcinoma (LUAD) by regulating cell cycle, DNA damage repair, and genome instability. LIN28B-AS1, identified as a CT-lncRNA, has the similar effect of oncogene with LIN28B. Downregulation of LIN28B-AS1 can decrease the expression of LIN28B, while the loss of LIN28B doesn't influence in the same way. Purified LIN28B-AS1 RNA-complex has found no evidence of direct binding between it and LIN28B, while a notable RNA-binding protein, IGF2BP1, has been detected by mass spectrometry (MS) analysis. The physical interaction between LIN28B-AS1 and IGF2BP1 was validated by RNA pull-down assays and IGF2BP1 could directly bind to LIN28B further affirmed by RIP assays. Inhibiting LIN28B-AS1 and IGF2BP1 in NCI-H1299 cells respectively and treating cells with actinomycin D potently shortened the half-life of LIN28B messenger RNA (mRNA) transcripts. Knocking down them both would have a stronger power to disturb the stability of mRNA of LIN28B. LIN28B-AS1 acted as a “scaffold” to carry IGF2BP1 to target to mRNA of LIN28B, and then stabilized it and finally participated in the progression of LUAD 21. The dysfunction of EGFR had been reported in many kinds of cancers 22-24, including renal cancer 25. The complementary transcript of EGFR, noted as EGFR-AS1, had drawn attention to the researchers to dig the deep relationship between EGFR and EGFR-AS1 in renal cancer. Both of them were upregulated and promoted the proliferation and invasion of Renal cell carcinoma (RCC) in vitro and in vivo 26. While EGFR-AS1 could directly bind the mRNA of EGFR to prevent it from degradation, it was also been proved that EGFR-AS1 could directly interact with HuR, also known as ELAVL1 27, which form a complex targeted to mRNA of EGFR and further stabilized it verified by RIP and Pull-down assays. Whether more molecules joining in the progress of interaction between EGFR-AS1 and EGFR needed broader and deeper exploration. He et al. found Long noncoding RNA FAM83A-AS1 enhanced the mRNA stability of FAM83A by binding with NOP58 and promoted the progression of Hepatocellular carcinoma (HCC) 28. Liu et al. elucidated that LEF1‐AS1 maintained LEF1 mRNA stability by binding to HNRNPL and enhanced the activity of the wnt signaling pathway to induce the cell proliferation, migration, or invasion of Osteosarcoma (OS) 29.

The interaction between antisense LncRNAs and the corresponding sense coding genes are not always synergistic and sometimes would manifest negative adjusting and controlling patterns. ZFPM2-AS1 was upregulated in LUAD and negatively modulate the expression of ZFPM2. To find out the cluster of proteins which may participate in this negative pattern, mass spectrometry (MS) analysis and the pull-down assays based on ZFPM2-AS1 had been carried out, and the team finally found UPF1 which had been a critical factor in RNA degradation pathways 30. It had been further proved that ZFPM2-AS1 and UPF1 directly bond the 3'UTR region of mRNA of ZFPM2 and shorten the half-life of it. Interestingly, no matter disturbing any one of ZFPM2-AS1 and UPF1, the formation of the complex would be negatively affected and insufficiently exert the function of RNA decay. All the evidences figured out that ZFPM2-AS1-UPF1-ZFPM2 axis were the stable pattern to promote proliferation, invasion, and EMT in LUAD 31.

## Alternatively spliced AS-lncRNAs exert bidirectional regulation towards sense mRNAs

Alternative splicing is a ubiquitous phenomenon in post-transcription regulation process, which would produce different RNA variants and unique isoforms. A great of evidences had proved that the products of alternative splicing events would have different or even opposing functions during aberrant biologic behaviors especially in tumors, such as proliferation, cell cycle, apoptosis, angiogenesis, drug-resistance, invasion and metastasis 32-34. Muscleblind-like-3 (MBNL3), a oncofetal splicing factor, was high expressed in hepatocellular carcinoma (HCC) compared with normal liver tissues, and modulated the alternative splicing of PXN antisense transcript 1 (lncRNA-PXN-AS1). MBNL3 was obbligato in tumorigenesis of HCC because of the nearly complete inhibition of subcutaneous colonization in nude mice. PXN-AS1, containing 5 exons, had two main transcript, PXN-AS1-001 (deleting exon 4, termed PXN-AS1-S) and PXN-AS1-002 (containing exon 4, termed PXN-AS1-L). MBNL3 recognized the intron 4 downstream of exon 4, promoted exon 4 inclusion of PXN-AS1 and finally upregulated the expression of PXN-AS1-L and decreased the expression of PXN-AS1-S through UV crosslinking and immunoprecipitation assays.

PXN-AS1-L could upregulate the protein level of PXN, while PXN-AS1-S had the opposite effect. To find out the underlying antagonistic mechanism, different deletion transcripts of PXN-AS1, exon 4 inclusion and exon 4 preclusion, was transiently transfected in SMMC-7721 cells, which exhibited stabilizing ability of prolonging the half-life of PXN mRNA only including exon 4. Even though PXN mRNA pull-down assays verified the direct interaction between PXN mRNA and the both of PXN-AS1-L and PXN-AS1-S, PXN-AS1-L, but not PXN-AS1-S, bond the PXN mRNA 3'UTR and inhibited miRNA-24-AGO2 complex-induced degradation of PXN mRNA in an exon-4-dependent manner. PXN-AS1-S and other transcripts that contained 5'region of exon 5 could robustly decrease the ribosome elongation rate on PXN mRNA through 5' region of exon 5 and the CDS of PXN mRNA direct binding. Interestingly, PXN-AS1-L had exon 4 and 5' region of exon 5 simultaneously and itself alone might not exert significant effect on PXN mRNA translation, which manifested that the coaction of PXN-AS1-L and PXN-AS1-S maintained the steady state in adult liver tissues until MBNL3 broke the balance by upregulating PXN-AS1-L and inhibiting PXN-AS1-S (Fig. 1D). Under the alternative splicing of PXN-AS1 by MBNL3 in HCC, PXN mRNA was protected and then the protein of PXN played significant role in tumorigenesis 35.

## Positive feedback loop in “AS-lncRNA-medium protein-sense gene” mode

ZEB1‐AS1 had been reported to participate in progression of several kinds of cancer types and played a pivotal role in tumorigenesis 36, 37. Triple‐negative breast cancer (TNBC), the most aggressive subtype of breast cancer, which lacked estrogen receptor, progestin receptor and Her2, was insensitive to chemotherapy and targeted therapy and as a result had a poor prognosis 38, 39. ZEB1‐AS1 and ZEB1 were boosted in TNBC, and directly bound ELAVL1 affirmed by pulldown silver staining assay and RIP assay. ZEB1‐AS1 leaded ELAVL1 to ZEB1 mRNA to protect it from degradation. ChIP assay disclosed that ZEB1 had a strong binding affinity with the part 1 (P1) of the ZEB1‐AS1 promoter. ZEB1 could bind the promoter of ZEB1‐AS1 and promoted the activation of the expression of ZEB1‐AS1 further corroborated by luciferase reporter assay. ZEB1‐AS1 and ZEB1 could interact with each other and combined with ELAVL1 to form a positive feedback loop to amplify the promoting effect on TNBC 40.

“AS-lncRNA-medium protein-sense gene” can product a stable mode to stimulate the expression of lncRNA and sense gene continuously. Cutting off the circle may be a new treatment mode for refractory and drug-fast cancers.

## AS-lncRNAs act as “ceRNA” to sequester miRNA to target sense genes

Abundant lncRNAs had been reported to act as competing endogenous RNAs to bind miRNAs respond element and upregulate target mRNAs 41. Actually, this category of lncRNAs interact with mRNAs in an indirect way by post transcriptional regulation as ceRNAs. LncRNA forkhead box P4 antisense RNA 1 (FOXP4-AS1) was upregulated in prostate cancer (PCa) and foreboded unfavorable patients' prognosis. As a nearby gene, FOXP4 also had an oncogenic property in PCa. Ago 2-RIP assay enriched both FOXP4-AS1 and FOXP4 and suggested that they might involve in a ceRNA network (Fig. 1C). MS2-RIP assay focused on two strongest affinity miRNAs, miR-3184-5p and miR-423-5p. As only miR-3184-5p could affected by the expression of FOXP4-AS1, luciferase assays further verified that it directly interacted with FOXP4-AS1 and FOXP4 mRNA. FOXP4-AS1 acted as a “sponge” to sequester miR-3184-5p to upregulate FOXP4 mRNA and promoted the malignant biological activity of PCa 42.

## Transcription Regulation Patterns of Antisense LncRNAs in Nucleus

LncRNAs existing in nucleus may have diverse functions including transcriptional regulation in cis or trans manner, organization of nuclear domains and so on. The common mechanism of them is that LncRNAs have the potential of interacting with chromatin-modulating proteins, facilitating their recruitment to chromatin, thereby controlling transcriptional activity 43. Antisense lncRNAs in nucleus would get an easier chance to modulate their neighboring sense genes by transcriptional regulation.

## AS-lncRNA regulate the state of H3K4me3 enrichment in promoter region of sense genes

A novel lncRNA, SATB2-AS1, consistent with SATB2, was recognized as a colorectal tissue specific lncRNA and was obviously higher than other tissues. SATB2-AS1 plays a pivotal role in modulating metastasis and the immune response of colorectal cancer (CRC); Therefore, the loss SATB2-AS1 promotes malignant progression in CRC. A series of rescue experiments verified that SATB2-AS1 fulfilled the function of CRC inhibition by regulating the expression of SATB2. The protein samples pulled down by biotinylated SATB2-AS1 and subjected to mass spectrometry analysis focused on WDR5 and GADD45A, a core subunit of the human histone H3K4 methyltransferase complex and a regulator of DNA demethylation respectively. The enrichment of H3K4me3 of the SATB2 promoter region was obviously lower in CRC cells than in normal colorectal tissues, which was predicted by ChIP-Seq data and verified by ChIP-qPCR. The decreasing expression of SATB2 caused by DNA hypermethylation and histone H3K4me3 loss in the promoter region because of the decreasing recruitment of WDR5 and GADD45A. ChIRP assays further affirmed the direct interaction between SATB2-AS1 and the promoter region of SATB2. SATB2-AS1, as a “scaffold”, recruited WDR5 and GADD45A to the promoter region of SATB2, which promoted DNA demethylation and histone H3K4me3 deposition, and inhibited CRC progression 44.

ZEB1‐AS1 was once reported primarily distributed in cytoplasm of triple‐negative breast cancer and bladder cancer 40; While it also could regulate transcription by mainly locating in nucleus in other cancers. In prostate cancer, ZEB1‐AS1 could promote proliferation and migration by directly binding to the region of ZEB1 promoter. ZEB‐AS1 directly bound MLL1, a major methyltransferase responsible for H3K4 trimethylation, to recruit it to the region of ZEB1 promoter, which promoted H3K4me3 deposition and upregulated the expression of ZEB1. It had been well recognized that ZEB1 could inhibit the expression of miR200c in transcriptional level and miR200 could further suppressed the expression of BMI1, as an oncogene associated with adverse pathologic and clinical features in prostate cancer 45. The loss of ZEB1 and ZEB1‐AS1 could potently induce the up-regulation of ZEB1‐AS1 and down-regulation of BMI1, which could rescued by the recovery of ZEB1 or ZEB1‐AS1. The ZEB1‐AS1-ZEB1- miR200c-BMI axis played a vital role in prostate cancer. The reason why the same AS-lncRNA can exist in cytoplasm or in nucleus of different types of cancers to exert disparate functions on its sense gene may be the result of different productions of variants by alternative-splicing process 9. The full-length of ZEB1‐AS1 detected here was 2535 nt. Which was 33 nt shorter than the data from NCBI database and even different from the 2449 nt in hepatocellular carcinoma detected by Li et al 37.

Zhang et al. found that EZR-AS1 upregulated EZR (variant 1) expression and promoted cell migration in esophageal squamous cell carcinoma (ESCC) tissues and cell lines. Because of the 4-fold abundance in nucleus compared with cytoplasm location, EZR-AS1 affected EZR promoter region -87/+134 and activated it affirmed by truncated promoter constructs luciferase reporter assay. Even though SP1 and AP-1 transcriptional factors was once reported to bind in EZR promoter region -87/+134, EZR-AS1 affected the activation of EZR promoter by forming a complex with RNA polymerase II, which was independent of SP1 and AP-1. SMYD3, a histone H3-lysine 4 (H3-K4)-specific methyltransferase, had been testified that it could upregulated the expression of EZR. ChIP assays suggested that SMYD3 protein bind from SBS-1(SMYD3 binding site 1) to SBS-4 in EZR promoter, and only SBS-1 and SBS-2 could affect SMYD3 and H3K4me3 enrichment under the condition of the change of EZR-AS1. Luciferase assays further verified that only containing SBS-1, the EZR promoter could be activated. Intriguingly, both pull-down and RIP assays clearly proved that only full-length EZR-AS1 and integral SMYD3 could interacted with each other. Finally, the increasing H3K4me3 deposition in EZR promoter activated the transcription of it 46.

## Conclusion

According to anatomical classification, antisense lncRNAs have inherent advantage to regulate the neighbor genes, especially for sense genes which have the overlapping regions on the opposite strand. AS-lncRNAs can directly bind to and stabilize sense genes mRNAs, recruit other proteins to prolong the half-life of target mRNAs, and even exert bidirectional function on opposite genes in cytoplasm (Fig. 1A, 1B, 1C and 1D). The mainstream mode in nucleus was that AS-lncRNAs regulate the methylation states of sense genes promoter region and activate or inactivate target genes (Fig. 1E). In this review, the typical AS-lncRNAs and sense genes regulation modes in different human cancers were listed and summarized in Table 1.

Current mechanism researches of lncRNAs are just the tip of the iceberg in the great field of noncoding transcriptome. With the deeper exploration, the clues of the regular regulation mode of antisense lncRNAs with sense genes on development of cancers will be clear and more meaningful. It would confer us more efficient means to learn human cancers and ways to treat malignant tumors.

## Figures and Tables

**Table 1:** List of typical regulation modes between AS-lncRNAs and sense genes in variant cancer types

Antisense lncRNA	Location	Sense gene	Description	Types of cancer	Reference
KRT7-AS	Cytoplasm	KRT7	KRT7-AS and KRT7 form an RNA-RNA duplex structure that protects KRT7 mRNA from RNase degradation.	Gastric cancer (GC)	11
MUC5B-AS1	Cytoplasm	MUC5B	MUC5B-AS1 formed an RNA-RNA duplex with MUC5B to increase MUC5B expression	Lung adenocarcinoma (LUAD)	16
MACC1-AS1	Cytoplasm	MACC1	Promoted MACC expression by Forming a RNA-RNA hybrid with it and recruiting Lin28 to it	Gastric cancer (GC)	18
LIN28B-AS1	Cytoplasm	LIN28B	Recruited IGF2BP1 to LIN28B mRNA and stabilize it	lung adenocarcinoma (LUAD)	21
EGFR-AS1	Cytoplasm	EGFR	Directly interacted with HuR to maintain EGFR stability	Renal cell carcinoma (RCC)	26
FAM83A-AS1	Cytoplasm	FAM83A	FAM83A-AS1 enhanced the mRNA stability of FAM83A by binding with NOP58.	Hepatocellular carcinoma (HCC)	28
LEF1‐AS1	Cytoplasm	LEF1	LEF1‐AS1 sponged HNRNPL which bond with LEF1 mRNA and stabilized it	Osteosarcoma (OS)	29
ZFPM2-AS1	Cytoplasm	ZFPM2	ZFPM2-AS1 and UPF1 directly bond the 3'UTR region of mRNA of ZFPM2 and shorten the half-life of it	Lung adenocarcinoma (LUAD)	31
PXN-AS1	Cytoplasm	PXN	One of the variants, PXN-AS1-L, bond the PXN mRNA 3'UTR and inhibited miRNA-24-AGO2 complex-induced degradation of PXN mRNA	Hepatocellular carcinoma (HCC)	35
ZEB1‐AS1	Cytoplasm	ZEB1	ZEB1-AS1 leaded ELAVL1 to ZEB1 mRNA to protect it from degradation and was affected by ZEB1 in its promoter region	Triple-negative breast cancer (TNBC)	40
FOXP4-AS1	Cytoplasm	FOXP4	FOXP4-AS1 acted as a “sponge” to sequester miR-3184-5p to upregulate FOXP4 mRNA	Prostate cancer (PCa)	42
SATB2-AS1	Nucleus	SATB2	Recruited WDR5 and GADD45A to the promoter region of SATB2, which promoted DNA demethylation and histone H3K4me3 deposition	Colorectal cancer (CRC)	44
ZEB1-AS1	Nucleus	ZEB1	Directly bound MLL1 and recruited it to the region of ZEB1 promoter, promoted H3K4me3 deposition and upregulated the expression of ZEB1	Prostate cancer (PCa)	9
EZR-AS1	Nucleus	EZR	Full-length EZR-AS1 and integral SMYD3 could interacted with each other, which increased H3K4me3 deposition in EZR promoter and activated the transcription of it.	Esophageal squamous cell carcinoma (ESCC)	46
